# Anti-proliferative and immunomodulatory properties of kaffir lime leaves and bioactive compounds on macrophages co-cultured with squamous cell carcinoma

**DOI:** 10.1371/journal.pone.0281378

**Published:** 2023-02-21

**Authors:** Thitiya Luetragoon, Yordhathai Thongsri, Krai Daotak, Pachuen Potup, Kanchana Usuwanthim

**Affiliations:** 1 Faculty of Allied Health Sciences, Department of Medical Technology, Nakhonratchasima College, Nakhonratchasima, Thailand; 2 Faculty of Allied Health Sciences, Cellular and Molecular Immunology Research Unit, Naresuan University, Phitsanulok, Thailand; Northwest University, UNITED STATES

## Abstract

Head and neck squamous cell carcinoma (HNSCC) is the seventh most common cancer worldwide. Late-stage patients have a significant chance of local recurrence and distant metastasis, as well as poor prognosis. Therapeutic goals for patients must be improved and personalized to reduce adverse effects. This study explored the anti-proliferative activity and immunomodulation potential of the constituents of crude kaffir lime leaf extract (lupeol, citronellal and citronellol) under co-culture. Results showed high cytotoxicity to human SCC15 cell line but not to human monocyte-derived macrophages. Treatment with crude extract and the contained compounds also suppressed cell migration and colony formation of SCC15 compared to the untreated control group, while high levels of intracellular ROS production were detected in the treatment group of SCC15. The Muse^TM^ cell analyzer revealed cell cycle arrest at G2/M phase and apoptosis induction. Inhibition of Bcl-2 and activation of Bax, leading to induction of the downstream caspase-dependent death pathway were confirmed by Western blot analysis. Co-culture with activated macrophages, kaffir lime extract and its constituents enhanced the development of pro-inflammatory (M1) macrophages and boosted TNF-α production, resulting in SCC15 apoptosis. Findings revealed novel potential activities of kaffir lime leaf extracts and their constituents in inducing M1 polarization against SCC15, as well as direct anti-proliferative activity.

## Introduction

Head and neck squamous cell carcinoma (HNSCC) was the seventh most common cancer worldwide in 2018 with 890,000 new cases and 450,000 deaths [1]. This cancer can arise from the mucosal epithelium in the oral cavity, oropharynx, hypopharynx and larynx [2]. HNSCC has been related to alcohol consumption, tobacco smoking, human papillomavirus (HPV) infection for cancers of the oropharyngeal region, the Epstein-Barr virus (EBV) associated with nasopharyngeal cancer and ultraviolet radiation from sunlight exposure for lip cancer [3].

Cancer is a complicated disease involving tumor microenvironment (TME) that defines the nature of cancer, not by the genetics of the tumor cells alone but by the surrounding environment that the tumor cells need for survival, growth, proliferation, invasion, and metastasis. The production of numerous intercellular mediators such as cytokines, chemokines and vesicles attract non-transformed cells in the surrounding area. The consequences are TME formation and close interaction with cancer cells to support the development of cancer hallmarks [4–6]. The TME comprises cancer cells and multiple normal cell types like fibroblasts, myofibroblasts and immune cells as well as cytokines, vascular tissue, and the surrounding extracellular matrix. Hence, complex interaction of different cellular signaling pathways with immune cell components in TME influences cancer initiation, progression, and therapeutic response [7,8].

Macrophages play an important role in the innate immune system by maintaining immune homeostasis. They are involved in several processes of initiating and regulating the immune responses to foreign antigens [9]. Two major polarization states have been described for macrophages as classically activated macrophages (M1 macrophages) that exhibit pro-inflammatory responses in host defense systems against a variety of bacteria, protozoa and viruses and play a role in anti-tumor immunity, while alternatively activated macrophages or M2 macrophages have anti-inflammatory functions and regulate tissue repair and remodeling [10,11]. In cancer, tumor-derived factors drive macrophages toward an immunosuppressive M2 marker following their recruitment to the tumor. They are called tumor-associated macrophages (TAMs) and have been linked to immune suppression and growth, angiogenesis, metastasis, and invasion of cancers [12,13]. M2-like TAMs can inhibit T cell function directly by multiple mechanisms and are associated with poor prognosis outcomes in solid tumors [14].

Macrophages are involved in tumor development and suppression. Immunostimulation of macrophages contributes to tumor regulation as a promising therapeutic target. Numerous studies have revealed that many plants or natural compounds have an immunomodulatory effect on macrophages. Methanolic extract from *Pouteria campechiana* leaves stimulated the proliferation of murine macrophages, phagocytic activity, nitric oxide (NO), hydrogen peroxide (H_2_O_2_) and cytokines (interleukin (IL)-6 and tumor necrosis factor (TNF)-α) production [15]. Crude polysaccharide extract from *Curcuma xanthorrhiza* Roxb. significantly increased phagocytosis and release of NO, H_2_O_2_, TNF-α and PGE2 in a dose-dependent manner in macrophages by specific activation of nuclear factor-κB (Nf-κB) [16]. In Japan, Ando *et al*. (2002) revealed that polysaccharides from safflower petals (*Carthamus tinctorius* L.) activated macrophages by recognizing and binding to the specific Toll-like receptor 4 on their surfaces [17]. Moreover, Nordin *et al*. investigated the immunomodulatory effects of *Clinacanthus nutans* extracts on THP-1 macrophages in co-culture with triple-negative breast cancer cells (MDA-MB-231). They concluded that this plant limited the pro-inflammatory condition in TME and showed potential anti-cancer properties on highly metastatic breast cancer condition [18].

*Citrus hystrix* DC., is commonly called makrut lime or kaffir lime. This plant belongs to the subfamily Aurantioideae, order Sapindales, family Rutaceae [19]. Kaffir lime leaves (KL) are a well-known herb used in Southeast Asian recipes and provide a unique and strong aroma. Lime leaves have historically been used as traditional medicine to treat ailments such as headache, flu, fever, sore throat, dizziness, bad breath and indigestion [20]. KL contain a variety of biologically active compounds including vitamin C, folic acid, potassium, flavonoids, tannins, saponins, glycosides, coumarins, pectins and dietary fibers [19]. Various volatile organic compounds found in KL include b-pinene, stearic acid, neophytadiene, limonene-oxide, copaene, linalool, caryophyllene, lupeol, tocopherol, dihydromyrcenol, citronella and citronellol [21]. The leaves of this plant possess hepatoprotective action against paracetamol-induced hepatotoxicity in rat [22]. KL exhibited antibacterial activity against Group A streptococcal and three respiratory pathogenic pathogens as *Staphylococcus aureus*, *Streptococcus pneumoniae* and *Haemophilus influenzae* [23,24]. In anti-cancer activity, KL has been studied for its cytotoxic effect against cervical cancer and neuroblastoma cell lines [25] and showed decreased leukemic cell proliferation [26,27]. Our previous study revealed that crude hexane extract, citronellol and citronellal inhibited growth of triple-negative breast cancer MDA-MB-231 cells by reducing cell proliferation and inducing cell cycle arrest and apoptosis [28].

However, the anti-proliferative potential on head and neck cancer and the immunomodulatory effect of KL on macrophages remain unknown. Here, the anti-proliferative properties of KL and its active compounds on squamous cell carcinoma 15 cell line (SCC15) and the immunomodulatory effect on THP-1 derived macrophages against SCC15 were investigated.

## Material and methods

### Preparation of kaffir lime leaf (KL) extracts and compounds

Powdered kaffir lime leaves Lot. No. 250818 were obtained from Khaolaor Laboratories Co., Ltd., Samut Prakan, Thailand. The method for KL extraction was described in our previous study [29]. The powdered leaves (1000 g) were sequentially macerated in hexane (3 L) for 3 days, followed by 95% ethyl acetate (3 L) for 3 days and 95% ethanol (3 L) for 3 days at room temperature and then filtrated. The filtrate was collected and evaporated by a rotary evaporator at 35°C. The maceration procedure for each solvent was carried out in duplicate. Three crude extracts were obtained as crude hexane (KL H), crude ethyl acetate (KL EtOAc) and crude ethanolic (KL ET) extracts. Lupeol was found in the crude ethanolic extract [29], while citronellal and citronellol were identified in the crude hexane extract [28]. Crude extracts (KL H and KL ET) were dissolved in 100% DMSO while compounds (lupeol, citronellol and citronellal) were dissolved in 50% DMSO and 50% Tween 80. In this study KL H, KL ET, lupeol, citronellal and citronellol (Sigma-Aldrich Inc., St. Louis, MO, USA) were used in all experiments.

### Monocyte isolation

Human monocyte-derived macrophages (MDM) were used as the primary normal cell control. Buffy coat was obtained from the Blood Bank, Naresuan University Hospital, Phitsanulok, Thailand. Ethics approval was obtained from the Human Ethics Committee of Naresuan University (IRB no. P1-0127/2565). Buffy coat was diluted with Hank’s balanced salt solution (HBSS) and then overlaid on 5 ml Lymphoprep (STEMCELL Technologies, Singapore) and centrifuged at 2000 rpm for 30 minutes. The mononuclear cell layer was collected and washed twice with HBSS buffer and the peripheral blood mononuclear cells (PBMC) were suspended in 5 ml of RPMI medium. Then, monocytes were separated by size sedimentation centrifugation using Percoll (GE Healthcare Bio-Sciences AB, Uppsala, Sweden) and the PBMC suspension was carefully overlaid on 10 ml of Percoll solution and centrifuged for 15 minutes at 2100 rpm. Monocytes between Percoll were collected and washed with HBSS, followed by centrifugation for 10 minutes at 1300 rpm. Isolated monocytes were cultured in RPMI 1640 and supplemented with 10% fetal bovine serum and 1% Antibiotic-Antimycotic purchased from Gibco^TM^ (Thermo Fisher Scientific, NY, USA). Cells were incubated at 37°C with 5% CO_2_ for 7 days until fully differentiate to macrophages, with media replacement every 3 days.

### Cell line and culture conditions

Squamous cell carcinoma (SCC) 15 (ATCC® CRL-1623™) was purchased from American Type Culture Collection (ATCC). Cells were cultured in Dulbecco’s modified Eagle’s medium and Ham’s F12 medium (Caisson Labs Inc., USA) containing 10% fetal bovine serum. Cells were incubated at 37°C with 5% CO_2_, with medium renewal every 2–3 days. The control conditions used untreated cells and Cisplatin (Sigma-Aldrich, MO, USA) as an anti-cancer positive condition.

### THP-1 cells and differentiation

THP-1 is a monocytic human leukemia cell line from the ATCC that can be differentiated into macrophages. This makes it possible to investigate, in co-cultures without direct cell contact interactions, how KL extracts and their constituents affect macrophage defense against cancer cells. THP-1 cells were maintained in RPMI 1640 Complete Medium supplemented with 10% FBS and 1% Antibiotic-Antimycotic (Thermo Fisher Scientific, NY, USA). Differentiation of the THP-1 cell line was induced by stimulation with 10 ng/ml of phorbol-12-myristate-13-acetate (PMA) (Sigma Aldrich, MO, USA) containing RPMI for 3 days, and the cells were incubated in fresh complete RPMI 1640 at 37°C with 5% CO_2_ for a further 2 days. THP-1-derived macrophages were cultivated with 20 μg/ml of lupeol, citronellal and citronellol and 100 μg/ml of crude hexane, and crude ethanol before being incubated for 24 hours. The phenotype of THP-1- derived macrophages was investigated by flow cytometry.

### Cellular cytotoxicity assay

MTT assay was used to determine the growth inhibitory role of crude extract and active compounds. MDM, THP-1-derived macrophage, and SCC15 were seeded in 96-well plates at a density of 1x10^4^ cells/50 μl/well. Cells were treated with various concentrations of compounds, lupeol, citronellol and citronellal (0.73, 1.46, 2.93, 5.86, 11.72, 23.44, 46.88, 93.75, 187.5, 375, 750, and 1,500 μg/ml). Moreover, cells were treated with serial dilutions of two crude extracts, KL hexane and KL ET at 4.88, 9.77, 19.53, 39.06, 78.13, 156.25, 312.5, 625, 1250, 2500, 5000, and 10,000 μg/ml. Cells were then incubated at 37°C for 24 hours. Fifty microliters of MTT (0.5 mg/ml) (Invitrogen, CA, USA) in medium free serum were added and cells were incubated at 37°C for 3 hours. MTT reagent was removed, and formazan crystals were dissolved in 100 μl of DMSO. Absorbance of the formazan solution was measured at 590 nm by a microplate reader (PerkinElmer Inc., MA, USA). This method followed the previous protocol [30]. The half-maximal inhibitory concentration (IC50) of KL H, KL ET, cisplatin, lupeol, citronellol and citronellal was calculated by dose-response relationships/sigmoidal curve fitting analysis. A 5% inhibitory concentration (IC5) was selected as non-toxic for cellular experiments.

### Cell cycle analysis

To confirm the growth inhibitory role of KL H, KL ET, lupeol, citronellol and citronellal, the cell cycle was analyzed using Muse™ Cell Cycle Kit (Merck, Darmstadt, Germany) following the manufacturer’s protocol. SCC15 cells were seeded in 24-well plates at a density of 1x10^4^ cells/well and incubated for 24 hours. The cells were treated with extracts and compounds for 24 hours. The SCC15 cell line from each experimental condition was harvested using trypsin/EDTA solution (Thermo Fisher Scientific, USA) and incubated at 37°C for 5 minutes. Then 200 μl of completed DMEM HamF12 was added to stop the reaction of trypsin. Cells were aspirated and centrifuged at 1500 rpm for 5 minutes and then fixed with 70% ethanol and incubated for at least 3 hours at -20°C. Cells were then washed twice with cold PBS and resuspended in 200 μl of Muse™ Cell cycle reagent before mixing gently and incubating for 30 minutes at room temperature in the dark. Cell cycle stage was then analyzed by a Muse™ Cell Analyzer.

### Cell apoptosis analysis

Muse™ Annexin V & Dead Cell Kit (Merck, Darmstadt, Germany) was used for apoptosis study. SCC15 cells from each experimental condition were harvested by trypsin/EDTA solution as described in cell cycle assay. Cells were washed in PBS and resuspended in the medium with 1% FBS. Then 100 μl of Muse™ Annexin V & Dead Cell Reagent was added, mixed gently and incubated for 20 minutes at room temperature in the dark. Cell apoptosis was measured using a Muse™ Cell Analyzer following the manufacturer’s protocol.

### Colony formation assay

Colony formation assay was used to study the potentiality of single cells to form colonies. This assay followed the previous description [31]. The SCC15 cell line was seeded into 6-well plates at a density of 500 cells/1.5 ml/well and incubated for 24 hours in standard culture conditions at 37°C. Cells were then treated with crude KL extracts, cisplatin, lupeol, citronellol and citronellal for 24 hours. The cells were incubated for 1 week with media replacement every 3 days. Cells were then fixed with 10% neutral buffer formalin solution for 30 minutes. The fixative reagent was removed, and the cells were stained with 2 ml of 0.5% crystal violet and incubated for 60 minutes at room temperature on a rotator. Cells were then washed 4 times in a stream of tap water. The plate was air-dried for at least 2 hours at room temperature. A 2 ml aliquot of methanol was then added to each well and the plate was incubated for 20 minutes at room temperature on a rotator. The optical density of each well was measured at 570 nm with a microplate reader (PerkinElmer Inc., MA, USA).

### Wound closure assay

Cell migration of the SCC15 cell line was evaluated by wound closure assay modified from a previous method [32]. The SCC15 cells were seeded into 6-well plates at a density of 1x10^6^ cells/ml and incubated at 37°C until reaching 80% confluence as a monolayer. The cell monolayer was scraped in a straight line with a SPL scar scratcher (SPL Life Sciences, Korea). Detached cells were removed and washed twice with 1 ml of medium. Cells were treated as described in the colony formation assay and incubated for 36 hours. A snapshot of cells from each condition was taken at time points of 6, 12, 24 and 36 hours using an inverted microscope (Carl Zeiss Microscopy GmbH, Germany). Distance of the wound area was analyzed by the ImageJ system.

### Intracellular ROS generation assay

H_2_DCFDA (Thermo Fisher Scientific, NY, USA) was used for detection of reactive oxygen species (ROS) production. Cells were seeded into dark clear bottom 96-well plates at 2.5 x 10^3^ cells/well and incubated for 24 hours in standard culture conditions at 37°C. The medium was removed, and the cells were incubated with diluted H_2_DCFDA solution (20 μM) for 45 minutes at 37°C in the dark. Next, cells were washed with PBS and treated with cisplatin, KL H, KL ET, lupeol, citronellol and citronellal for 3 hours. The plate was measured immediately on a fluorescence plate reader at Ex/Em = 485/535 nm in end point mode.

### SDS-PAGE and Western blot analysis

SCC15 were cultured with 12.5 μg/ml of cisplatin as a positive control. Cells were treated with each compound (lupeol, citronellal and citronellol), crude hexane and crude ethanol at 100 μg/ml. Total proteins of the SCC15 cell line from each condition were extracted by ice-cold RIPA lysis buffer (Bio Basic Inc., NY, USA) in the presence of Halt Protease/Phosphatase Inhibitor Cocktails (Thermo Fisher Scientific, NY, USA), and centrifuged at 12,000 rpm for 15 minutes at 4°C. Quantification of total protein concentration was performed by the Bradford Coomassie-binding colorimetric method. The protein extract was mixed with an equal volume of 4X Leammli loading buffer and heated to denature at 95°C for 5 minutes. Samples were loaded into wells of 12% SDS-polyacrylamide gel electrophoresis (PAGE), and proteins were separated according to molecular weight and transferred to a polyvinylidene fluoride membrane (Bio-Rad Laboratories Inc., Hercules, CA, USA). For Western blot analysis, the membrane was blocked overnight at 4°C with blocking buffer containing 5% bovine serum albumin (Capricorn Scientific GmbH, Germany) in Tris-buffered saline with Tween 20 (TBST) buffer. The membrane was blotted using primary antibodies specific to cleaved-caspase 3 (Asp175, p17) (Affinity Biosciences, OH, USA), β-actin, pro-caspase 3, Bax and Bcl-2 (Santa Cruz Biotechnology Inc., Dallas, TX, USA) for one hour at room temperature on a rotator. The membrane was then washed with TBST and incubated with horseradish peroxidase-conjugated goat anti-mouse IgG (H+L) secondary antibody (Thermo Fisher Scientific, NY, USA) for one hour at room temperature. The membrane was observed by soaking in chemiluminescence substrate for 5 minutes and placed in a ChemiDoc XRS+ Imaging System (Bio-Rad Laboratories Inc., Hercules, CA, USA). The chemiluminescence signal of the blotted membrane was detected by Image Studio Lite software (LI-COR Corporate, Lincoln, NE, USA).

### Flow cytometry

Cell surface markers were analyzed by immunostaining with PE-conjugated anti-human CD80 and FITC-conjugated anti-human CD163 (BioLegend, San Diego, CA, USA). Cells were suspended with FACS buffer solution and directly stained with fluorescence-conjugated antibody. The reaction was incubated at 4°C for 1 hour in the dark and the cells were then washed twice with PBS. The cell pellet was resuspended with staining buffer and analyzed by an FC 500 Flow Cytometer (Beckman Coulter Inc., Indianapolis, IN, USA). FITC-conjugated mouse IgG1,κ isotype control and PE-conjugated mouse IgG1,κ isotype control (BioLegend Inc., San Diego, CA, USA) were used as the negative control.

### Macrophage-tumor cell co-cultures

In the co-culture experiments, THP-1 monocytes were differentiated in 24-transwell inserts (SPL Life Sciences Co., Ltd., Korea). The THP-1 monocytes (2 × 10^4^ cells/well) were seeded into the upper chamber of the transwell apparatus. THP-1 monocytes were differentiated into macrophages by 3 days of incubation with 10 ng/ml of phorbol 12-myristate 13-acetate (PMA, Sigma-Aldrich). Cells were then washed, and the fresh complete RPMI was replaced for a further 2 days. THP-1-derived macrophages were cultivated with 20 μg/ml of lupeol, citronellal and citronellol and 100 μg/ml of crude hexane, and crude ethanol. SCC15 were seeded in the lower well at 5 × 10^4^ cells/well for 24 hours to allow their adherence to the walls. The chambers with the THP-1-derived macrophages were washed and then placed directly on top of the well plates containing the SCC15 cells, and the resulting co-culture systems were incubated for 24 hours. SCC15 was collected for cell apoptosis analysis using Muse™ Cell Analyzer.

### Evaluation of cytokine

Culture supernatants from differentiated macrophages in the insert wells of each experimental condition were harvested for determination of TNF-α by sandwich ELISA assay, following the manufacturer’s protocol (Sino Biological Inc., Pennsylvania, PA, USA). A 96-well plate was coated with capture antibody specific to TNF-α at 4°C for one night. The plate was washed four times with a wash buffer between each step. The plate was then blocked with blocking solution for 1 hour with shaking. Supernatant from the cell culture of each condition and the standard were placed into the reaction and shaken for 2 hours at room temperature. The plate was washed, followed by binding of 100 μL of detection antibody, and incubated for 1 hour before washing. Avidin horseradish peroxidase-conjugated secondary antibody was then added, and the plate was shaken for 30 minutes. The reaction was completed by adding 100 μL of freshly mixed solution of 3,3’,5,5’-tetramethylbenzidine (TMB). After incubating for 15 minutes in the dark, the stop solution was added. The absorbance of the reaction was measured at 450 nm using an EnSpire® Multimode microplate reader (PerkinElmer Inc., MA, USA). The production of cytokines in each condition was calculated from standard curves using known concentrations of recombinant cytokines.

### Statistical analysis

All experimental conditions were triplicated to provide accurate results. One-way ANOVA and the Bonferroni multiple comparisons test were used for data analysis by GraphPad Prism software. A confidence interval of 95% (*p* = 0.05) was used in all statistical analyses.

## Results

### Cellular cytotoxicity of crude extracts and lupeol, citronellal and citronellol on MDM, THP-1 and SCC15

**Fig 1:**
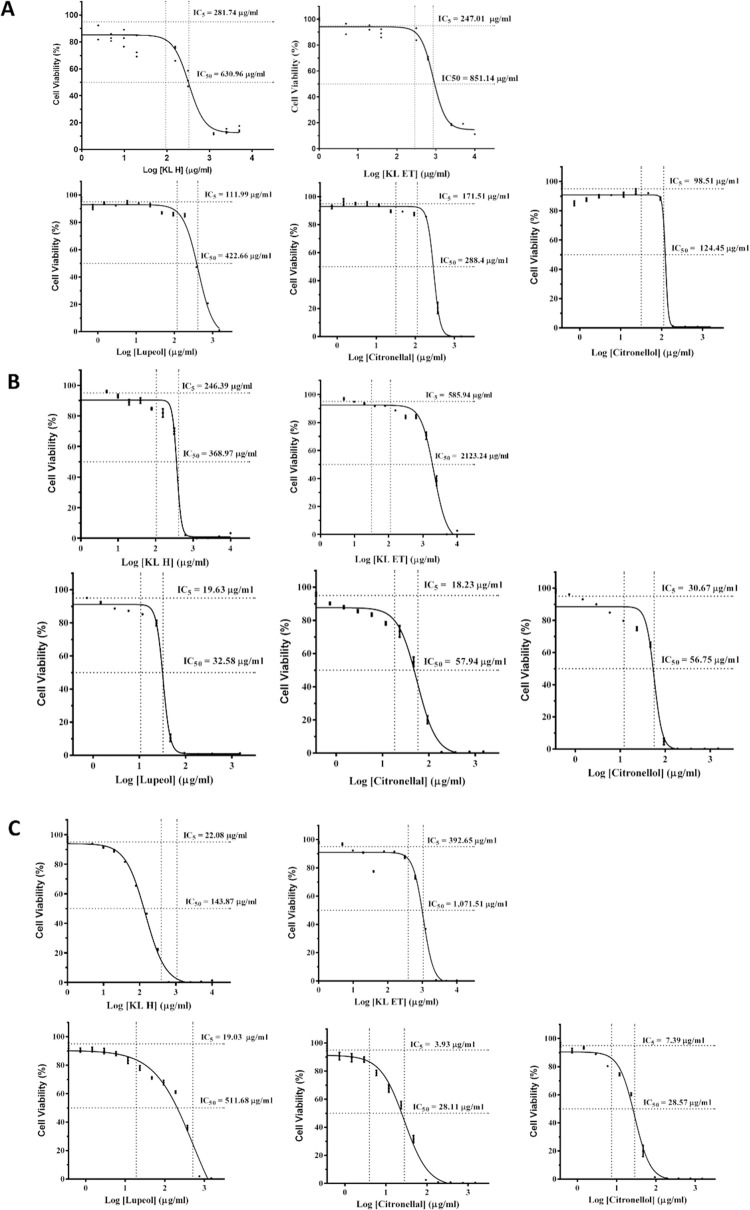
Investigation of the effect of KL H, KL ET, lupeol, citronellal and citronellal on cell viability of human monocyte-derived macrophages (MDM), THP-1 and SCC15 cell lines. MTT assays were performed after cell treatment for 24 hours. (A) IC5 and IC50 values of MDM after treatment under different concentrations of lupeol, citronellal and citronellal. (B) IC5 and IC50 values of extracts and compounds on THP-1. (C) IC5 and IC50 results for anti-proliferative effect of KL H, KL ET, lupeol, citronellal and citronellal on SCC15. KL H: Crude hexane; KL ET: Crude ethanolic extract.

MTT was performed to evaluate the optimal dose of anti-proliferative effect of crude extract (KL H and KL ET) and active compounds (lupeol, citronellol and citronellal). Human monocyte-derived macrophages (MDM), SCC15 and THP-1 were treated with different concentrations of extracts and compounds for 24 hours. IC5 values of lupeol, citronellal and citronellol on human MDM were 112 μg/ml, 171.5 μg/ml and 98.5 μg/ml, respectively (Fig 1A) and higher than those on SCC15 (Fig 1C). These non-toxic optimal doses were used for cell culture treatment. Cellular cytotoxicity of crude extracts and compounds on THP-1 was also measured to evaluate the optimal dose for THP-1 monocyte differentiation and polarization. The IC5 values of KL H, KL ET, lupeol, citronellal and citronellol were 246.39 μg/ml, 585.94 μg/ml, 19.63 μg/ml, 18.23 μg/ml and 30.67 μg/ml, respectively (Fig 1B).

### Effect of crude extracts and their active compounds on cell cycle distribution of SCC15 cell line by Muse™ Cell Analyzer

**Fig 2:**
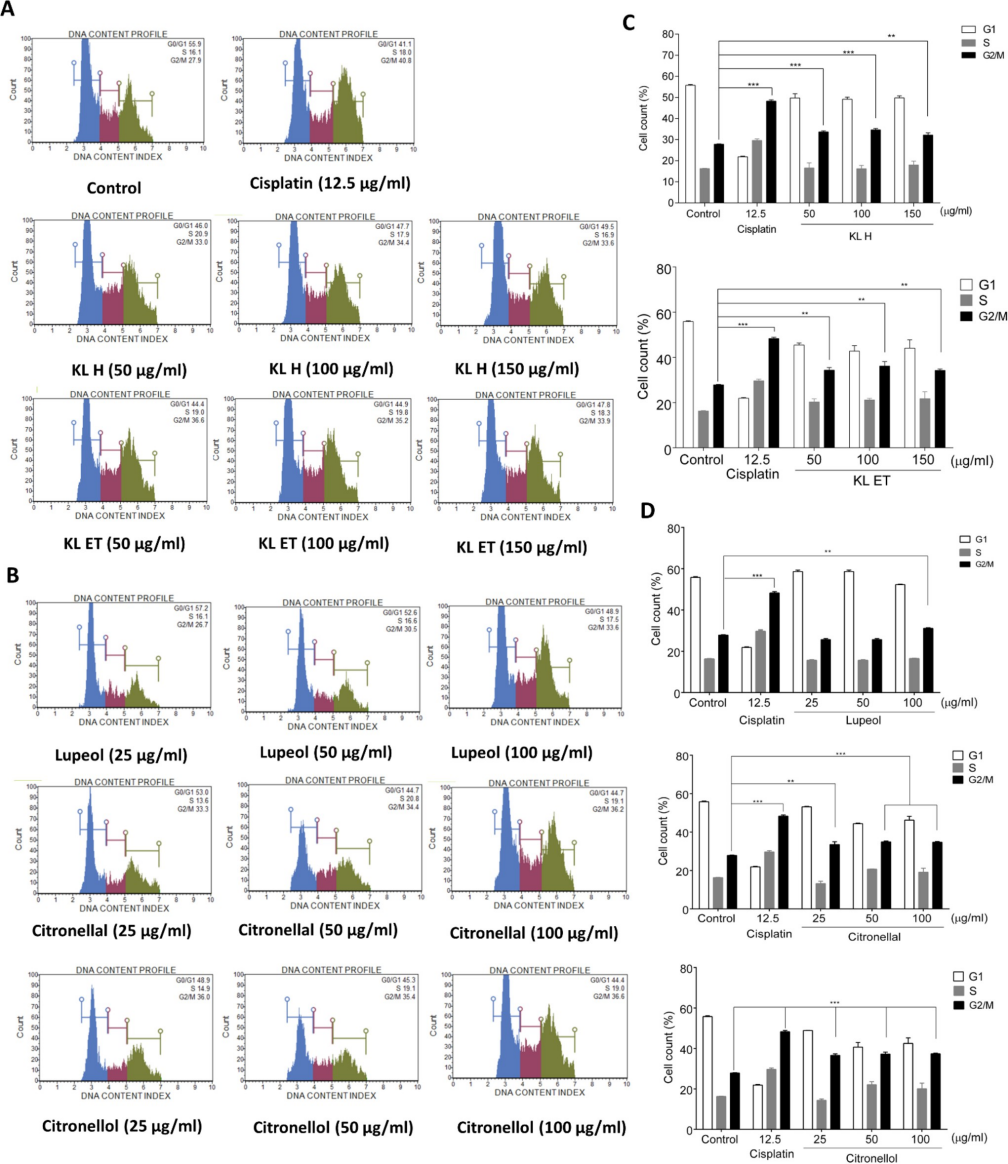
Efficacy of KL H, KL ET, lupeol, citronellal and citronellol on the distribution of SCC15 in the cell cycle analyzed by a Muse™ Cell Analyzer. (A), and (B) DNA content index histograms of cell populations in each phase of the cell cycle in SCC15 cell line after treatment with cisplatin, crude extracts and compounds for 24 hours. (C) and (D) show the percentage of cells in G1, S and G2/M phase for the SCC15 cell line. Data are presented as means ± SEM. **Statistically significant (*p* ≤ 0.01) and ***p ≤ 0.001 compared to the control. KL H: Crude hexane; KL ET: Crude ethanolic extract.

Cell cycle assay was assessed by a Muse™ Cell Cycle Kit and nuclear DNA of the cell line was intercalated with propidium iodide (PI). Cells were discriminated at different phases of the cell cycle based on differential DNA content including G0/G1, S and G2/M phase. To investigate the effect of the extracts, lupeol, citronellal and citronellol on cell cycle progression, untreated and treated SCC15 were investigated by a Muse Cell Analyzer. DNA content index histograms of the control and treatment groups with crude extract and cisplatin in each cell cycle phase (G0/G1, S and G2/M) are shown in Fig 2A. In the treatment groups, KL H and KL ET significantly induced cell cycle arrest at the G2/M phase when compared to the control group (Fig 2A and 2C).

After treatment with lupeol, citronellal and citronellal, G2/M enrichment significantly increased in SCC15 compared to the untreated control (Fig 2B and 2D). This result indicated that crude extracts (KL H and KL ET), lupeol, citronellal and citronellol significantly induced cell cycle arrest at the G2/M phase in SCC15.

### Apoptosis induction effect of crude extracts and bioactive compounds on SCC15

**Fig 3:**
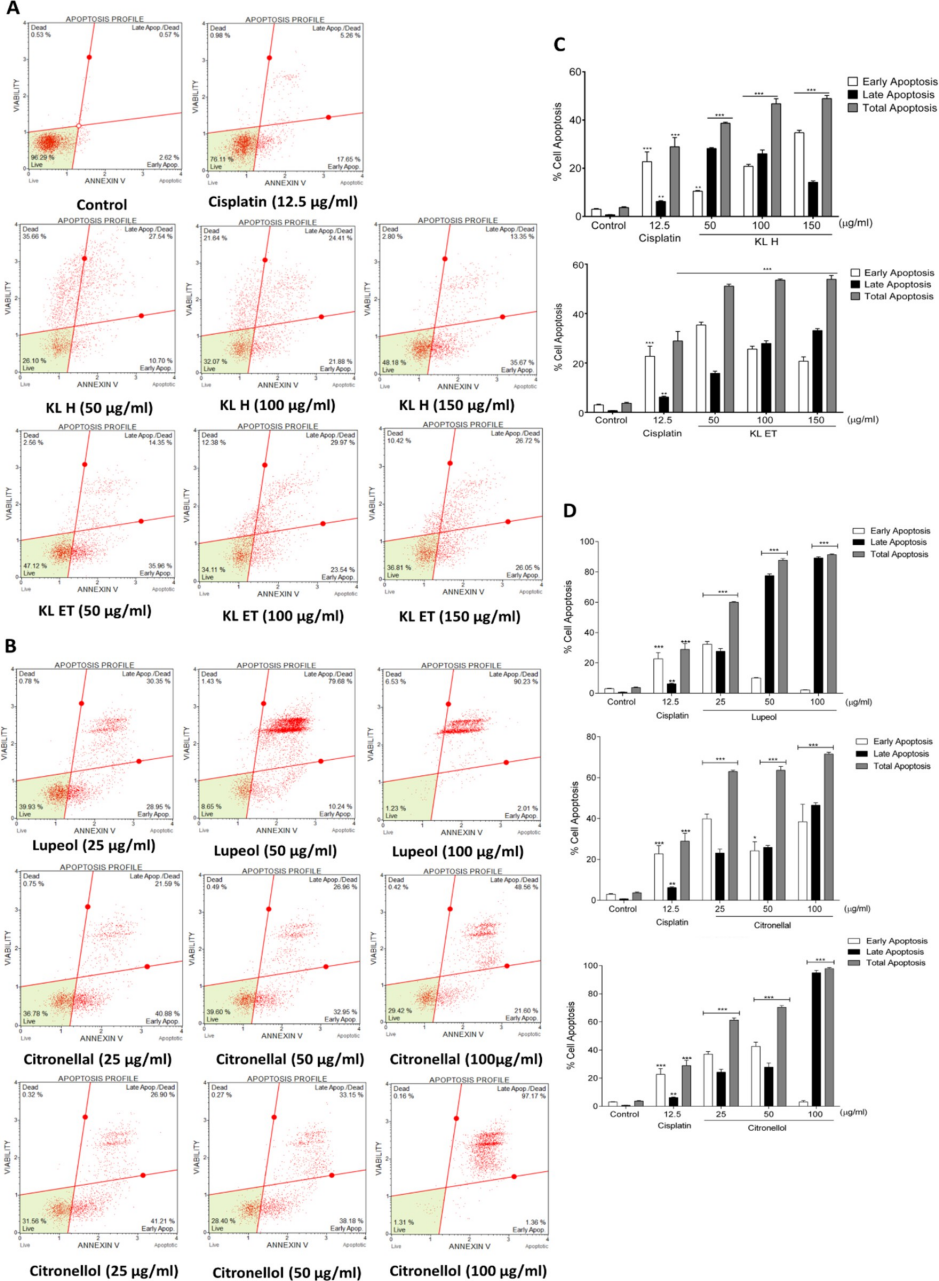
Determination of apoptosis in SCC15 cell line after treatment with KL H, KL ET, lupeol, citronellal and citronellol for 24 hours. (A) and (B) Dot plots of annexin V and 7-AAD dual staining showing the percentage of cell populations in each quadrant. (C) and (D) Bar graphs showing quantitative data of the percentage of early, late and total apoptotic cells. Results are presented as means ± SEM. **Statistically significant (*p* ≤ 0.01) and ***p ≤ 0.001 compared to the control. KL H: Crude hexane; KL ET: Crude ethanolic extract.

Induction of apoptosis in the SCC15 cell line was investigated after 24 hours of treatment with KL H, KL ET, lupeol, citronellal and citronellol. Apoptosis assay was performed using a Muse^TM^ Cell Analyzer following the Muse™ Annexin V & Dead Cell Kit procedure for the SCC15 cell line stained with annexin V and 7-AAD (7-amino-actinomycin D). The first and second quadrants represented dead cells and late apoptotic cells, respectively, while the third and fourth quadrants represented live cells and early apoptotic cells, respectively (Fig 3A and 3B). These dot plots revealed that all crude extracts and active compounds triggered early and late apoptosis in the SCC15 cell line, the same as the positive control, cisplatin. Statistical analyses of early, late and total apoptotic cells were represented as bar graphs. Results showed significantly increased percentage of early apoptosis after treatment with KL H, while treatment with KL ET, lupeol, citronellal and citronellol induced late apoptosis compared to the untreated control (Fig 3C and 3D). Findings suggested that treatment with crude KL extracts and their compounds strongly enhanced apoptosis in the SCC15 cell line.

### Crude KL extracts and bioactive compounds inhibited colony formation of SCC15 cell line

**Fig 4:**
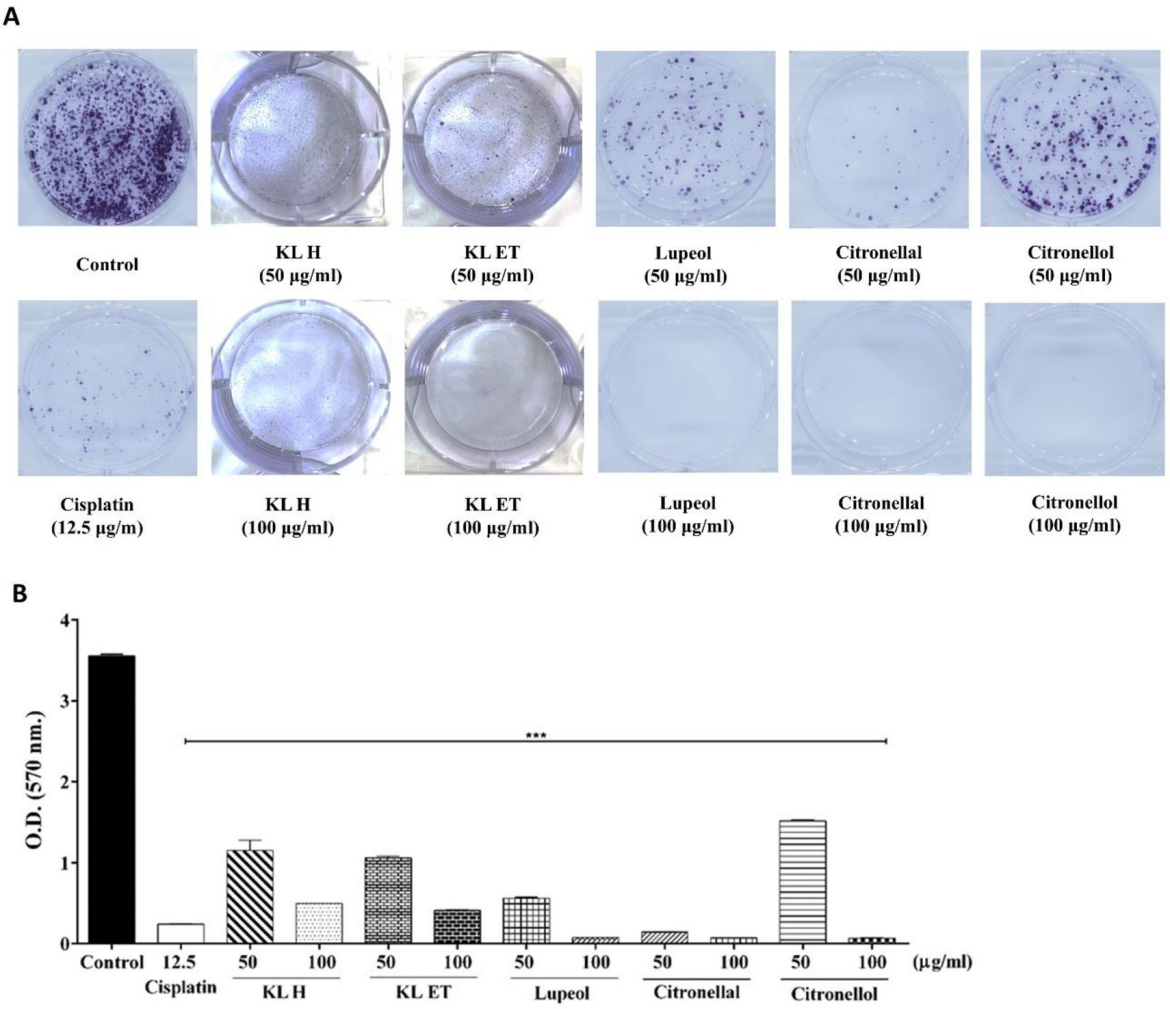
Colony formation of SCC15 cell line performed in 6-well plates, with cells stained with crystal violet. (A) Colony formation of untreated control and cells treated with cisplatin, crude extracts and compounds for 24 hours. (B) Colony quantification was measured by a microplate reader at OD 570 nm. Data are presented as means ± SEM. ***p ≤ 0.001 compared to the control. KL H: Crude hexane; KL ET: Crude ethanolic extract.

To study the growth of SCC15 cell line from a single cell to form colonies in the presence or absence of crude KL extracts and their bioactive compounds, SCC15 cell lines were seeded into 6-well plates and treated with different doses of substances. Results showed that the crude extracts and compounds suppressed colony formation of SCC15, the same as cisplatin treatment (Fig 4A and 4B). These data confirmed that KL H, KL ET, lupeol, citronellal and citronellol reduced colony formation in a dose-dependent manner, which is an important factor in cancer survival and progression.

### Crude KL extract and bioactive compounds inhibited migration of SCC15

**Fig 5:**
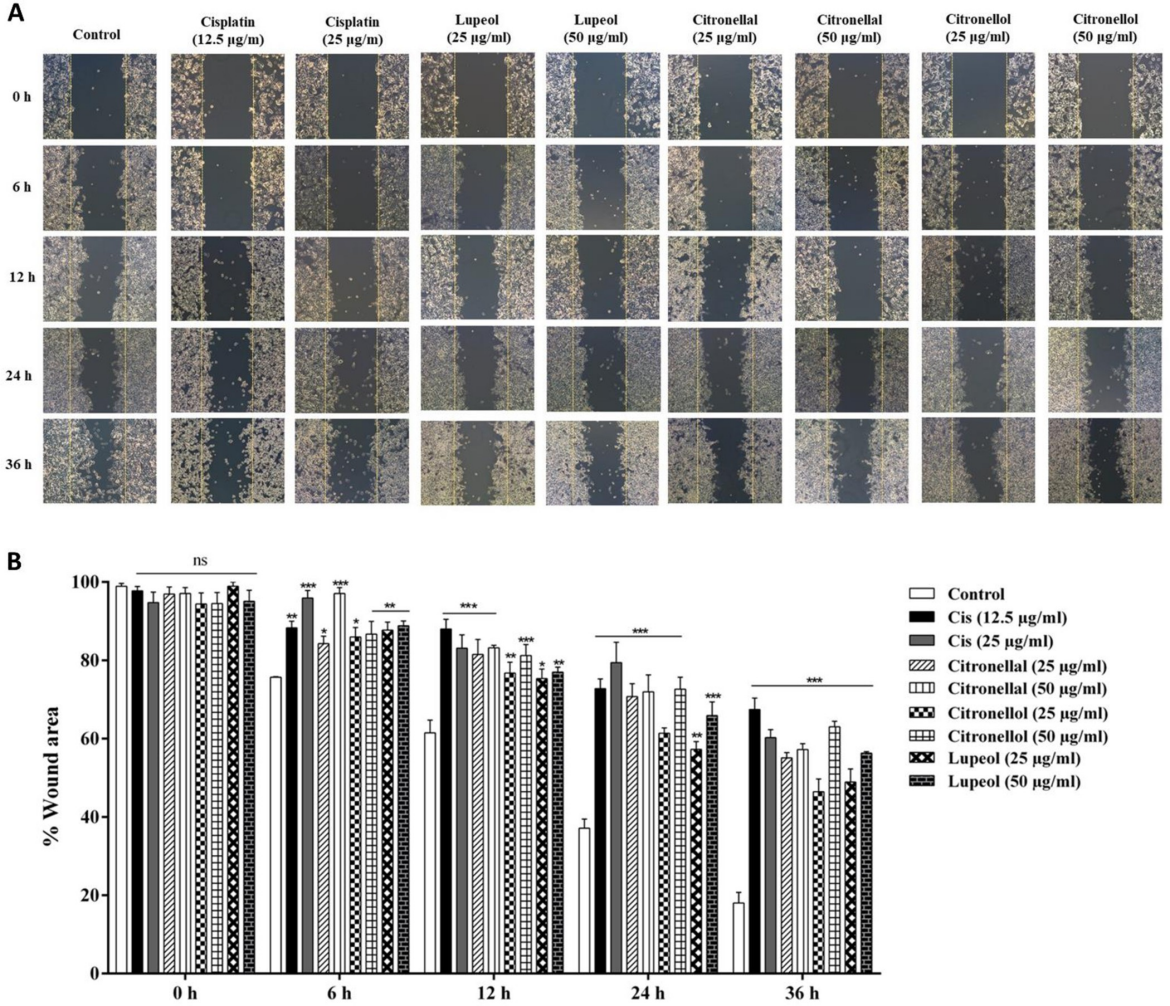
Cell motility assay of SCC15 cell line for untreated control and cells treated with crude extracts and their contained compounds for 36 hours. (A) Pictures of wound area taken at 0, 6, 12, 24 and 36 hours by an inverted microscope. (B) Distance of wound area measured using the ImageJ system. Percentage of wound area was calculated, with bar graphs representing means ± SEM. ns = not significant, * Statistically significant (*p* ≤ 0.05), ** *p* ≤ 0.01 and ***p ≤ 0.001 compared to the control. KL H: Crude hexane; KL ET: Crude ethanolic extract.

The wound closure assay is a method of *in vitro* study to analyze the migration of cell populations. Cell monolayers were scratched at similar size at 0 hours. The migrative ability of SCC15 was evaluated after treatment with cisplatin, crude extracts and their contained compounds. Results showed significant inhibition of cell migration after treatment at 6 hours. Percentage of wound area for cells treated with cisplatin, crude extracts and their contained compounds was significantly higher than the control. At 36 hours after wound induction, wound area of the untreated control was almost closed (18.02±4.69%), while wound areas of treated cells with drugs (67.42±5.09%) and all extracts and compounds were widely open, with percentage range 46.45±5.64 to 63.01±2.42% (Fig 5A and 5B).

### Intracellular ROS production in SCC15

**Fig 6:**
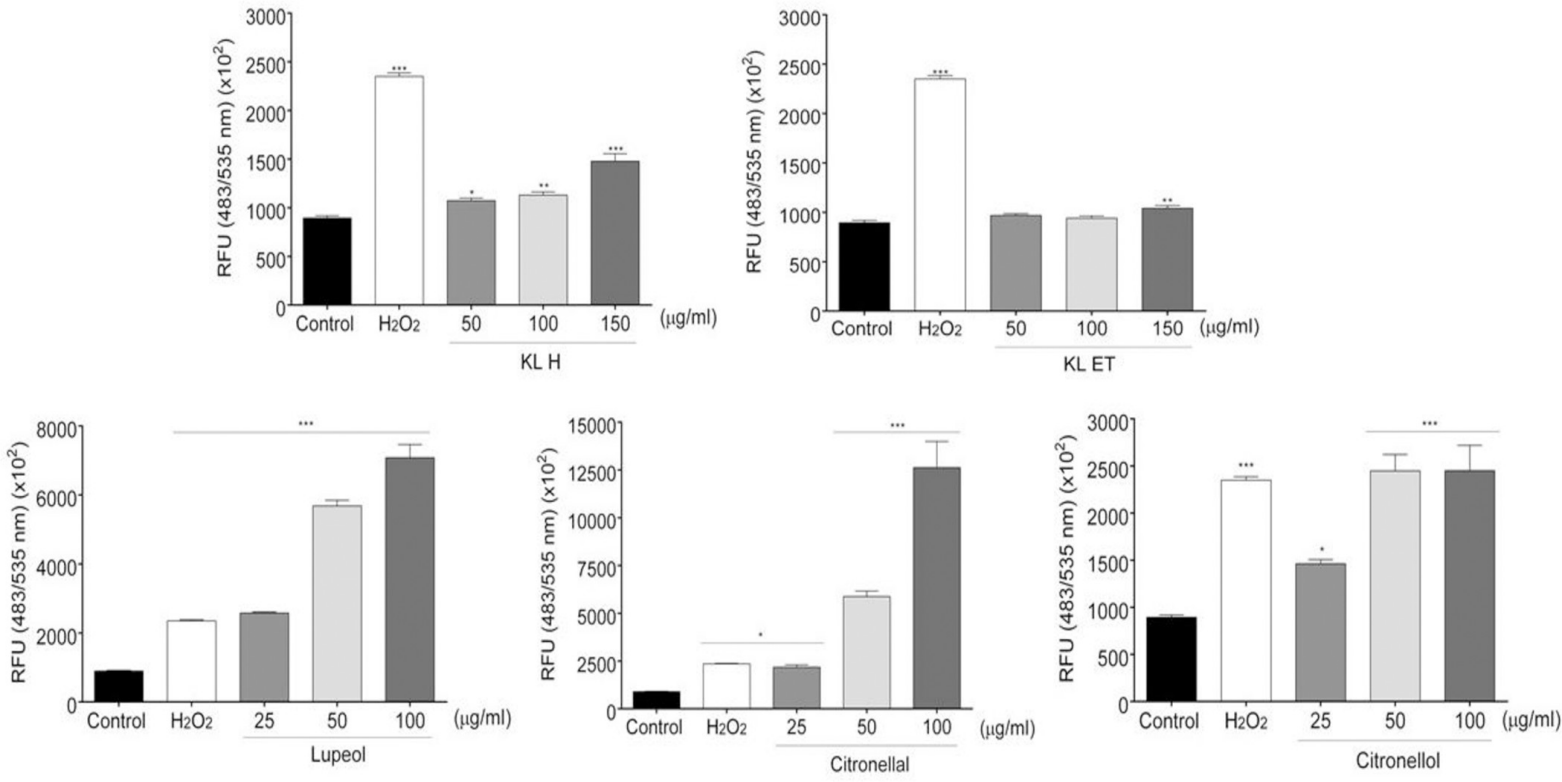
ROS generation of SCC15 cell line after treatment with extracts and active compounds for 3 hours. Cells were measured by a fluorescence plate reader at Ex/Em = 485/535 nm in end point mode. Bar graphs of relative fluorescence units (RFU) are represented as means ± SEM. * Statistically significant (*p* ≤ 0.05), ** *p* ≤ 0.01, and ***p ≤ 0.001, compared to the control. KL H: Crude hexane; KL ET: Crude ethanolic extract.

To determine the effect of compounds on ROS generation, SCC15 cells were treated with extracts and active compounds. Hydrogen peroxide, H_2_O_2_ (100 μM) was used as the positive control. Results showed that KL H, lupeol, citronellal and citronellol strongly induced ROS production in the SCC15 cell line. Interestingly, lupeol and citronellal stimulated high levels of ROS compared to the positive control (H_2_O_2_) in a dose-dependent manner. However, a low level of ROS was observed in the KL ET treatment. This finding indicated that high intracellular ROS generation was stimulated by crude extracts and their bioactive compounds (Fig 6).

### KL extracts and their active compounds induced apoptotic cell death in SCC15 cell line

**Fig 7:**
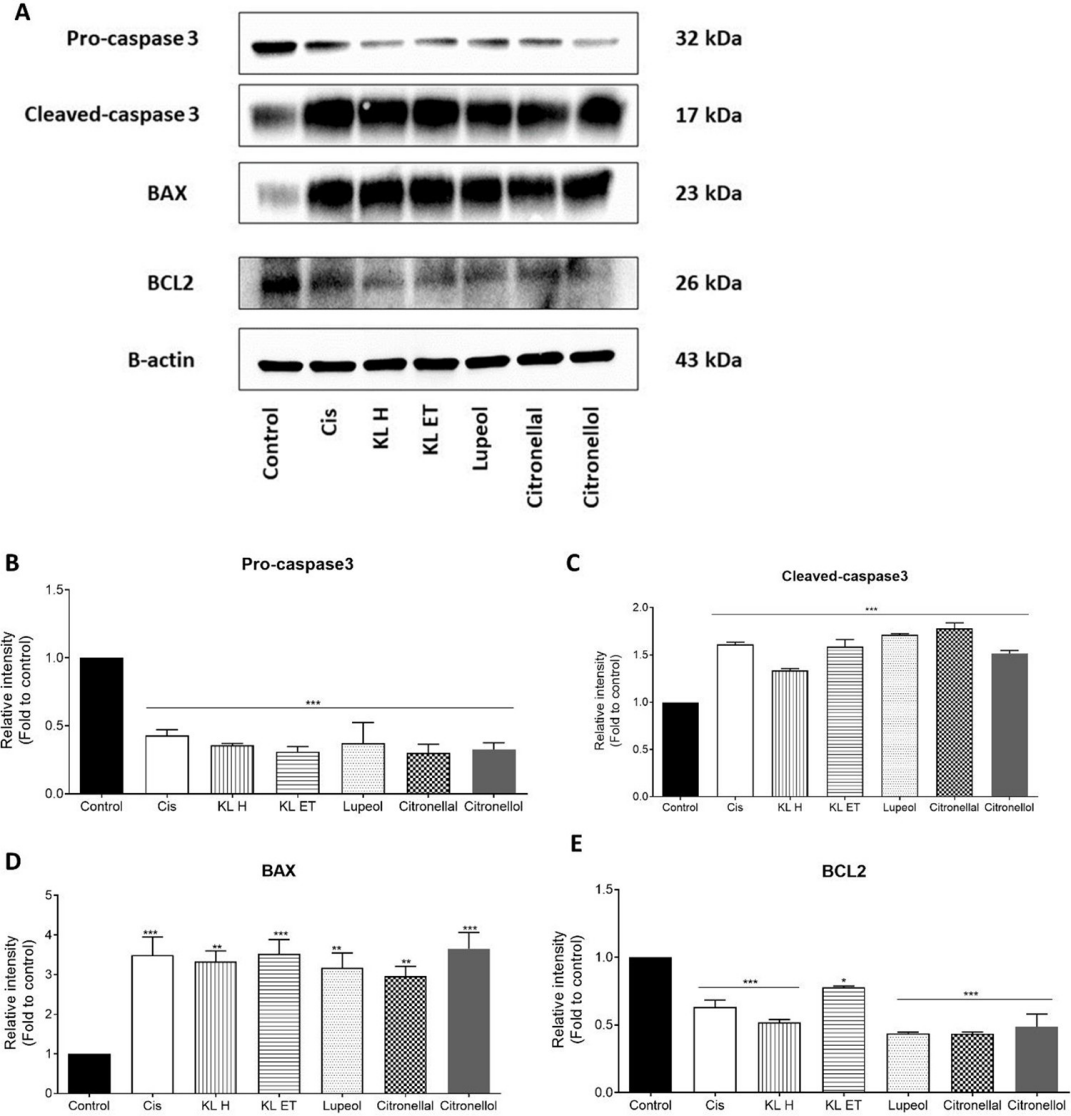
Western blot analysis of apoptotic proteins in SCC15 cells. (A) Band intensity of total protein levels of pro-caspase 3, cleaved-caspase 3, Bax and Bcl-2 in the control and treatment groups. (B) Relative intensity of pro-caspase 3, (C) cleaved-caspase 3, (D) Bax and (E) Bcl-2 were quantified by scanning densitometry and normalized to the control group. Bar graphs are represented as means ± SEM. *p ≤ 0.05, ** *p* ≤ 0.01 and ***p ≤ 0.001 compared to the control. Control: Untreated SCC15; Cis: Cisplatin; KL H: Crude hexane; KL ET: Crude ethanolic extract; Lu: Lupeol; Lal: Citronellal; Lol: Citronellol.

SCC15 were cultured with 100 μg/ml of lupeol, citronellal, citronellol, crude hexane, and crude ethanol. Western blot was performed to assess the expression of apoptotic-related proteins including pro-caspase 3, cleaved-caspase 3, Bax and Bcl-2. Results demonstrated that all crude extracts (KL H and KL ET) and active compounds (lupeol, citronellal and citronellol) significantly increased the levels of pro-apoptotic, Bax and cleaved-caspase 3. Treatment of SCC15 with KL extracts and their compounds resulted in suppression of Bcl‑2 expression and decreased pro-caspase 3, showing similar results to the positive drug control, cisplatin (Fig 7). These findings suggested that KL extracts and their bioactive compounds induced apoptosis in the SCC15 cell line by activation of Bax and cleaved-caspase 3.

### Effects of extracts and their compounds on THP-1 macrophage polarization

**Fig 8:**
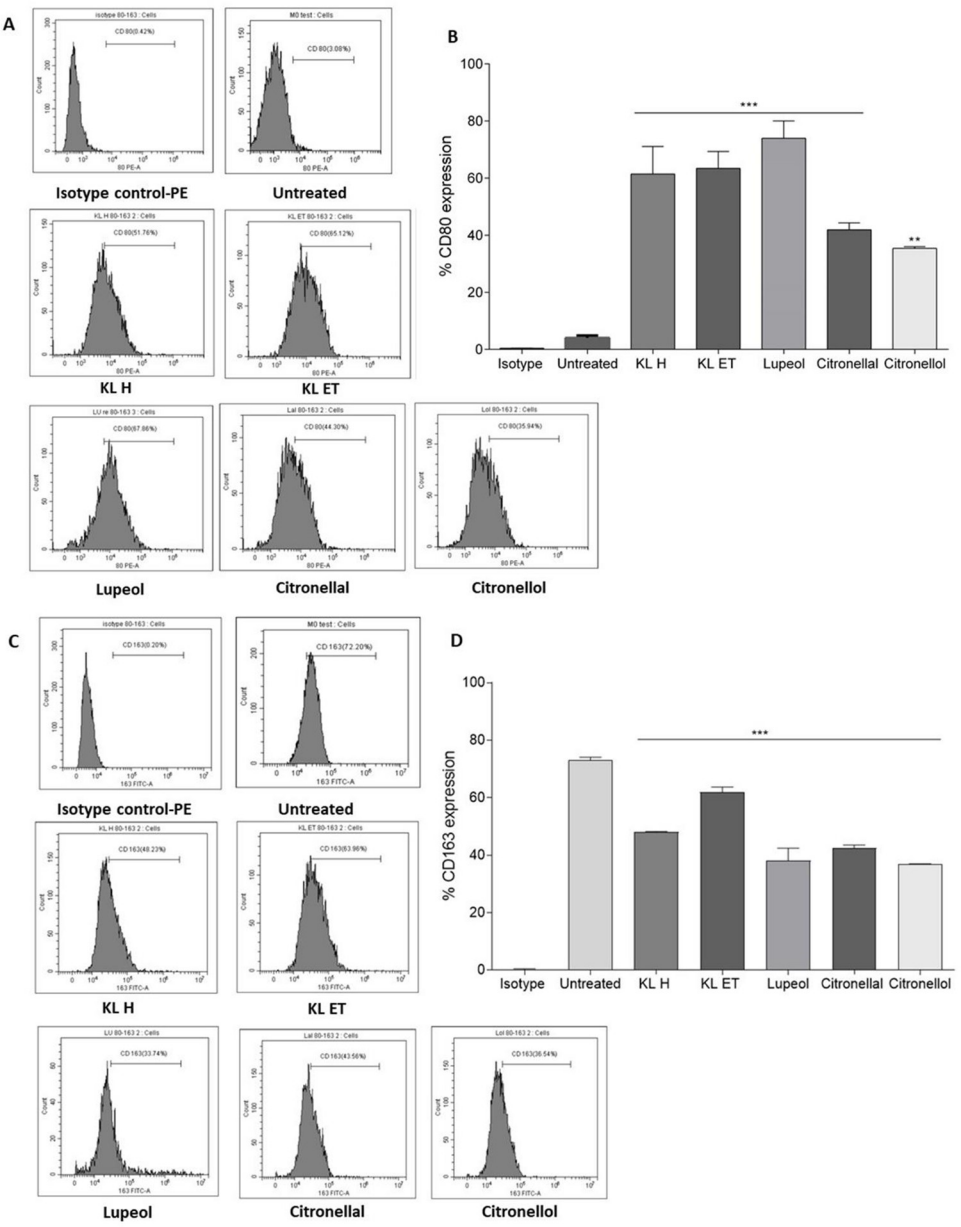
Characterization of the phenotype of THP-1 derived macrophages after treatment with crude KL extracts and their compounds. (A) and (B) Flow cytometry analyses of M1-typical surface markers representing the percentage of CD80 positive cells. (C) and (D) Expression of CD163 and M2 positive cells. Bar graphs are shown as means ± SEM., ***p ≤ 0.001, compared to the control.

M1 macrophages express nitric oxide synthase (iNOS), TNF-α, IL-6, CD80 and CD86 costimulatory molecules. By contrast, M2 macrophages were defined based on the co-expression of CD163 (scavenger receptor) and CD206 (mannose receptor) as anti-inflammatory markers and arginase-1 (ARG-1) [33,34]. We next questioned whether the KL extracts and the active compounds could polarize macrophages toward M1 or M2 types. To clarify the polarization phenotype of THP-1-derived macrophages, then cells were cultivated with 20 μg/ml of lupeol, citronellal and citronellol and 100 μg/ml of crude hexane, and crude ethanol before being incubated for 24 hours. Flow cytometry was performed to determine M1 and M2 positive cell populations. Flow cytometry analysis revealed that the number of CD80 positive cells was significantly higher in macrophages treated with KL extracts and their compounds compared to the untreated control (Fig 8A and 8B). By contrast, cell populations of CD163 decreased in the treatment group (Fig 8C and 8D).

### Effect of KL extracts and their compounds on cytokine secretion of THP-1 derived macrophages and cancer cell apoptosis in co-culture experiments

**Fig 9:**
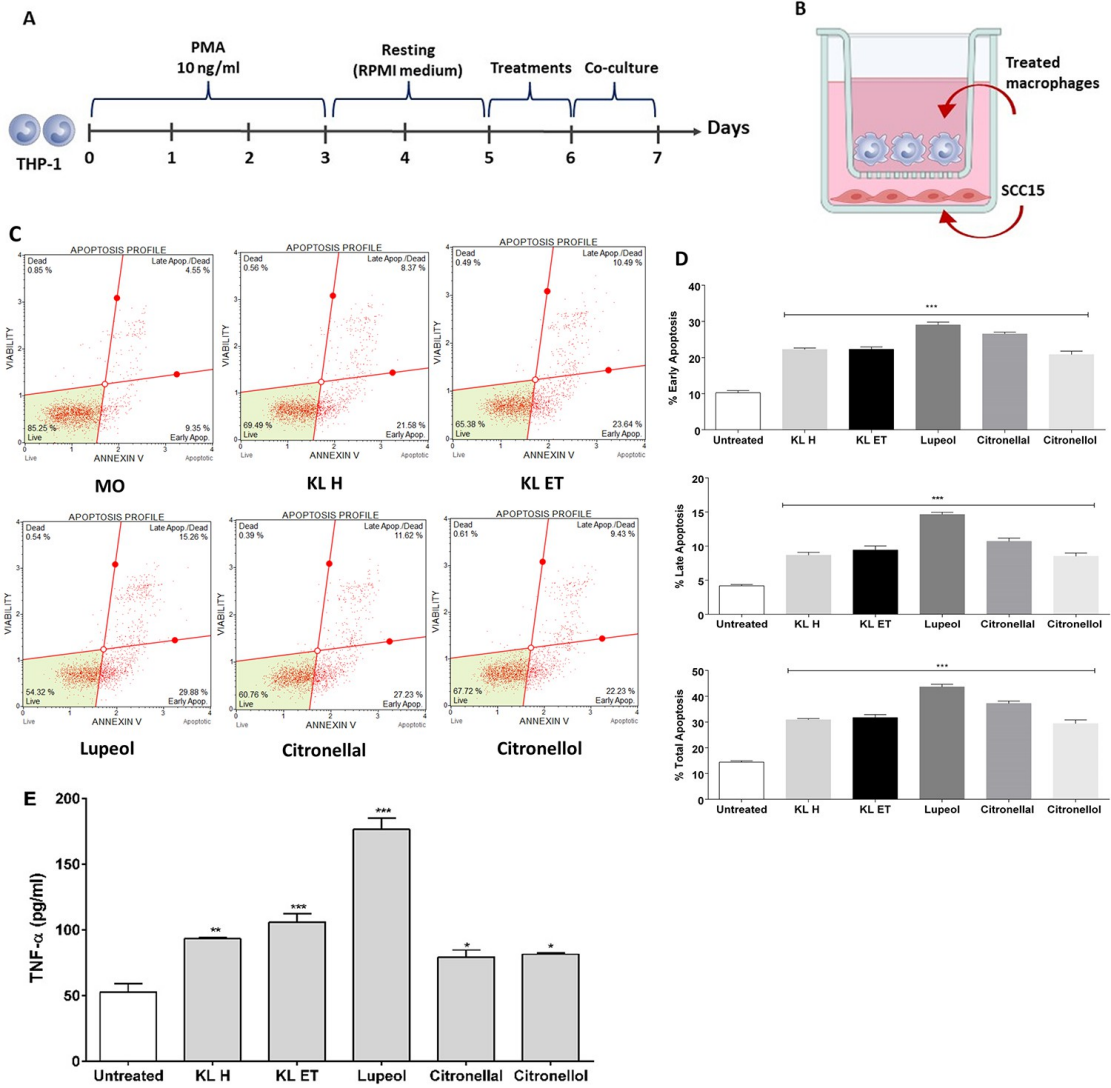
SCC15 co-cultured with differentiated macrophages. (A and B) Schematic diagram for THP-1 differentiation and co-culture condition in transwell plates. (C and D) SCC15 apoptosis analysis after co-culture with crude KL extracts and their compound treated macrophages. (E) TNF-α concentrations in the supernatants of macrophages harvested from the upper chamber. Bar graphs are represented as means ± SEM. *p ≤ 0.05, ** *p* ≤ 0.01 and ***p ≤ 0.001 compared to the untreated control (M0).

Crude KL extracts and their compounds (lupeol, citronellal and citronellol) polarized THP-1-derived macrophages toward M1. Next, we investigated the effect of crude extracts and compound-activated macrophages involved in anti-cancer activity. In this experiment, THP-1 cells were placed in the upper chamber and stimulated with PMA, followed by resting in fresh complete RPMI for 2 days. THP-1-derived macrophages were cultured with 20 μg/ml of lupeol, citronellal and citronellol and 100 μg/ml of crude hexane, and crude ethanol before being incubated for 24 hours and co-cultured with SCC15 at day 6, as shown in Fig 9A and 9B. Cell apoptosis assay was performed using a Muse^TM^ Cell Analyzer. Activated macrophages significantly induced cell apoptosis of SCC15 in the co-culture condition (Fig 9C and 9D). The release of cytokines (TNF-α) was evaluated in cell culture media using an ELISA assay in treated macrophages. Results showed that TNF-α highly increased in the extracts and their compound-exposed macrophages in the co-culture condition (Fig 9E). Collectively, data suggested that inhibition of cancer cell growth was mediated by TNF-α produced by M1-type pro-inflammatory macrophages.

## Discussion

Head and neck squamous cell carcinoma (HNSCC) is the seventh most common cancer worldwide. The incidence of HNSCC continues to rise and is anticipated to increase by 30% by 2030 [3]. In patients with early stages of the disease with no clinical nodal involvement, cure rates of over 80% can be achieved with single modality intervention. The principal modalities of curative therapy for locally confined HNSCC are surgery, radiation, and systemic therapy. Unfortunately, two-thirds of the patients are diagnosed at an advanced locoregional stage [35]. Moreover, current conventional treatment regimens for HNSCC have adverse effects [36]. Therefore, continuing research has been undertaken to find less-toxic therapies to reduce treatment morbidity for head and neck cancer. Many plants have shown tumor‑inhibitory activities on HNSCC such as curcumin [37], *Dioscorea nipponica* (dioscin) in SCC15 [38], *Rheum undulatum* L. in HN22 and SCC15 [39] and cannabidiol in FaDu, SNU899, SCC15, Hep2 [40]. In this study, crude kaffir lime leaves (KL) extracts and their compounds (lupeol, citronellal and citronellol) showed strong anti-proliferative potential as a cytotoxic drug, cisplatin. Moreover, they induced cytotoxicity in SCC15 but not in normal cells and human MDM, as shown in Fig 1.

Our previous study explored *Moringa oleifera* Lam., extract and compound 3-HBI inhibited cell proliferation and induced apoptosis in SCC15 cell line through the activation of cleaved caspase-3 and Bax as well as suppressing anti-apoptotic factor, Bcl-2 [41]. Crude extracts and compound-mediated apoptosis in SCC15 significantly increased the level of pro-apoptotic (Bax) and cleaved-caspase 3 and suppressed the expression of Bcl‑2 as anti-apoptotic. Anuchapreeda *et al*. suggested that the cell cycle of K562 (chronic myelocytic leukemia) was significantly arrested at the G2/M phase after treatment with a subfraction of kaffir lime leaves for 24 hours [27]. In this study, our findings were confirmed in a different cancer cell line. Crude KL extracts and their compounds (lupeol, citronellal and citronellol) inhibited tumor growth by induction of cell cycle arrest at G2/M phase and cell apoptosis in SCC15 same as a positive control drug, cisplatin. Cisplatin is one of the chemotherapeutic agents for treating the head and neck squamous cell carcinoma (HNSCC) patient. This drug can cause deoxyribonucleic acid (DNA) damage, blocking cell division and resulting in apoptotic cell death in cancer cells [42]. Our current results highlighted the strong anti-proliferative effect of KL extract, lupeol, citronellal and citronellol like chemotherapeutic drug, cisplatin (Figs 2 and 3).

Excessive increase in intracellular ROS induced DNA damage, cell cycle arrest and senescence as well as apoptosis of tumor cells [43]. Our study results demonstrated that intracellular ROS accumulation in SCC15 significantly increased in the treatment group, as shown in Fig 6. Data indicated that crude KL extracts and their compounds triggered apoptosis and induced G2/M phase cell cycle arrest associated with ROS accumulation. Uncontrolled cell growth, invasion and metastasis are all critical hallmarks of cancer [5]. Wound closure assay has been used to measure migration of individual cells in the leading edge of the scratch [32]. Colony formation assay or clonogenic assay was used to study the ability of single cells to grow into colonies [31]. Our results from wound closure assay and colony formation revealed that crude extracts of KL and their compounds reduced cancer cell migration and colony formation in a dose-dependent manner.

Since immunotherapeutic strategies that target macrophages in the treatment of cancer, particularly HNSCC, are a potential approach. We thus evaluate the immunomodulation properties of kaffir lime leaf extracts and their constituents on inducing macrophage polarization against SCC15 in addition to the direct anti-cancer activity of these substances. Conventional classifications of macrophage polarization include naive macrophages, which rapidly differentiate into the other 2 main phenotypes of classically activated macrophages (M1) and alternatively activated macrophages (M2). In most investigations on macrophage polarization, straightforward *in vitro* techniques were used. In general, some cytokines activate M1 or M2 polarization in macrophages generated from *in vitro* culture. Nitric oxide synthase (iNOS), TNF-α, IL-6, and the costimulatory molecules CD80 and CD86 were expressed by M1 macrophages. In contrast, M2 macrophages were identified based on the expression of the anti-inflammatory markers arginase-1 and CD163 (scavenger receptor and mannose receptor) [44]. Macrophages are a major component of the leukocytes present in the tumor microenvironment [45]. Due to their closeness to cancerous tumor cells, tumor associated macrophages (TAMs) are regarded as being essential cells in the disease. They may display different phenotypes depending on the microenvironment [46]. M1 macrophages promote inflammatory response against pathogens and tumor cells, while M2 macrophages play roles as immunosuppressive phenotypes [10]. Various cell types are present in the tumor site microenvironment. Macrophages as M2-like TAMs stimulate multiple mechanisms, thereby leading to immune suppression and tumor growth, angiogenesis, metastasis, and invasion. Tumor microenvironments containing higher proportions of M1-like TAMs initiate adaptive immune responses and prevent tumor development [12,13]. Therefore, regulating TAMs and maintaining their homeostasis has become the focus of tumor therapy.

In this study, we investigated whether polarization of macrophages toward M1 or M2 types might be induced by KL extracts and active compounds. Cells were cultured with kaffir lime leaf extracts and their constituents lupeol, citronellal, and citronellol in order to better understand the polarization phenotype of macrophages. The number of CD80 positive cells was significantly larger in macrophages treated with extracts and their components compared to the untreated control, according to flow cytometry used to identify M1 and M2 positive cell populations (Fig 8). The findings show that extract can direct macrophages toward the M1 target. Therefore, we next investigated into how the M1 polarized macrophages affected anti-SCC15 activity by co-culture model. THP-1 cells were placed in the upper chamber and stimulated with PMA, followed by resting in fresh complete RPMI for 2 days. THP-1-derived macrophages were cultured with 20 μg/ml of lupeol, citronellal and citronellol and 100 μg/ml of crude hexane, and crude ethanol before being incubated for 24 hours and co-cultured with SCC15 at day 6. The TNF-α was evaluated in cell culture media using an ELISA and cancer cell apoptosis was performed. Result show that TNF-α highly increased in the extracts and their compound-exposed macrophages in the co-culture condition while activated macrophages significantly induced cell apoptosis of SCC15 in the co-culture condition (Fig 9C and 9D). Since, TNF-α stimulated inflammation and also induced apoptosis [47,48]. Collectively, data suggested that inhibition of cancer cell growth was mediated by TNF-α produced by M1-type pro-inflammatory macrophages activated by those extract. These results demonstrated that THP-1 differentiated M1 macrophages were induced by KL extracts and their bioactive compounds and released high levels of TNF-α that were secreted into the cell supernatant, leading to death occurring in SCC15. Thus, KL possessed the function to send signals and activate M1 macrophages. However, targeting macrophages in cancer immunotherapeutic approaches is still challenging. Gao *et al*. reported that phospholipase D4 (PLD4) were involved in the activation process of M1 phenotype macrophages and developed antitumor effects in colon cancer cells [49], while another study demonstrated that ruyiping extract (RYP) reduced lung metastasis in triple-negative breast cancer by regulating macrophage polarization through reducing signaling transducer and activator of transcription (STAT) 6 [50]. Kaushik *et al*. showed that cold plasma treatment stimulated the differentiation of pro-inflammatory (M1) macrophages to prevent solid cancer progression via release of anti-cancer cytokines [51], while Nordin *et al*. concluded that *Clinacanthus nutans* extracts reduced pro-inflammatory condition in the co-culture of triple-negative breast cancer cells (MDA-MB-231) and THP-1 macrophages [18].

The Nf-κB and the STAT pathways are known to play pivotal roles in the transcriptional profile of macrophages. Among the transcriptional factors, STAT1 and canonical Nf-κB (p50/p65 heterodimer) were essential for M1 tumoricidal functions and triggered the expression of pro-inflammatory cytokines [52]. LPS is a well-known activator of the Nf-κB pathway and was important in the establishment of the M1 phenotype of macrophages. Further studies could investigate whether KL and its bioactive compounds activate STAT1 and Nf-κB.

Our findings highlighted the potential of crude KL extracts and their compounds lupeol, citronellal and citronellol in stimulating M1 type polarization against cancer via TNF-α induced apoptosis. However, further studies could investigate whether KL and its bioactive compounds activate STAT1 and Nf-κB. It is suggested to use a variety of surface markers and confirm TAM activity by comparing it to a positive control in order to trigger apoptosis due to the limits of our work to apply biomarkers for the M1 and M2 phenotype. Different HNSCC cancer cell types must validate the extract’s direct action, and these cells must also test various doses and time periods. The use of patient-derived xenograft models and HNSCC patient-derived samples in future studies will be very effective in examining combination effects and overcoming resistance mechanisms.

## Conclusions

**Fig 10:**
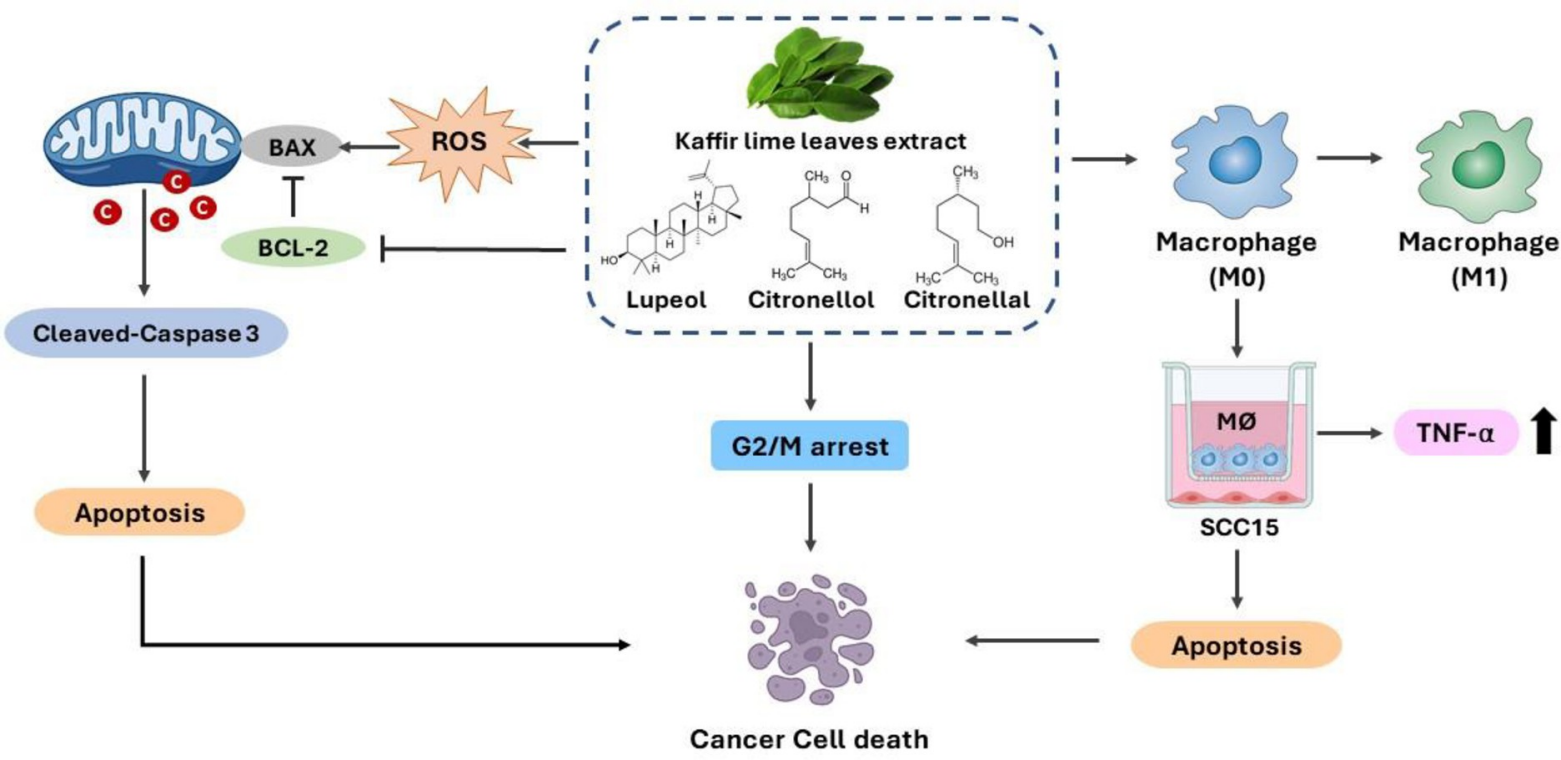
Anti-proliferative and immunomodulatory activity of kaffir lime leaf (KL) extracts and their bioactive compounds. KL extracts and the contained compounds induced G2/M cell cycle arrest. Apoptosis was stimulated by the treatments with the extracts and active compounds through ROS production, increasing BAX and cleaved-caspase 3 expression and decreasing the level of Bcl-2. The plant extracts and contained compounds also polarized macrophage into M1 type and increased the secretion of TNF-α, leading to apoptosis of SCC15 in co-culture with macrophages.

This study explored the anti-proliferative effects among KL extracts from various solvents as hexane and ethanolic extracts and their contained compounds including lupeol, citronellal and citronellol on the SCC15 cell line. This plant extract and compounds inhibited cell growth and triggered apoptosis through activation of the pro-apoptotic Bax and inhibition of the anti-apoptotic Bcl-2, thereby inducing the downstream caspase-dependent apoptosis pathway, caspase-3 in SCC15. Treatment with KL extracts and their contained compounds significantly increased cell cycle arrest at the G2/M phase of SCC15. They were also ROS inducers associated with high apoptosis and G2/M enrichment. Interestingly, this was the first report of the immunomodulatory effect of crude KL extracts and their contained compounds that polarized M1-macrophages and increased TNF-α production, resulting in apoptosis of SCC15 in co-culture with stimulated macrophages (Fig 10). Thus, bioactive compounds from KL extracts showed promise as an effective therapeutic agent for head and neck cancer treatment.

## Supporting information

S1 Raw images (PDF)
