# Supplementary material for: Anti-proliferative and immunomodulatory properties of kaffir lime leaves and bioactive compounds on macrophages co-cultured with squamous cell carcinoma
Source: PLoS One. 2023 Feb 21;18(2):e0281378. doi: 10.1371/journal.pone.0281378 (PMC9943011; doi:10.1371/journal.pone.0281378)
Supplement: S1 Raw images — (PDF) [file pone.0281378.s001.pdf]

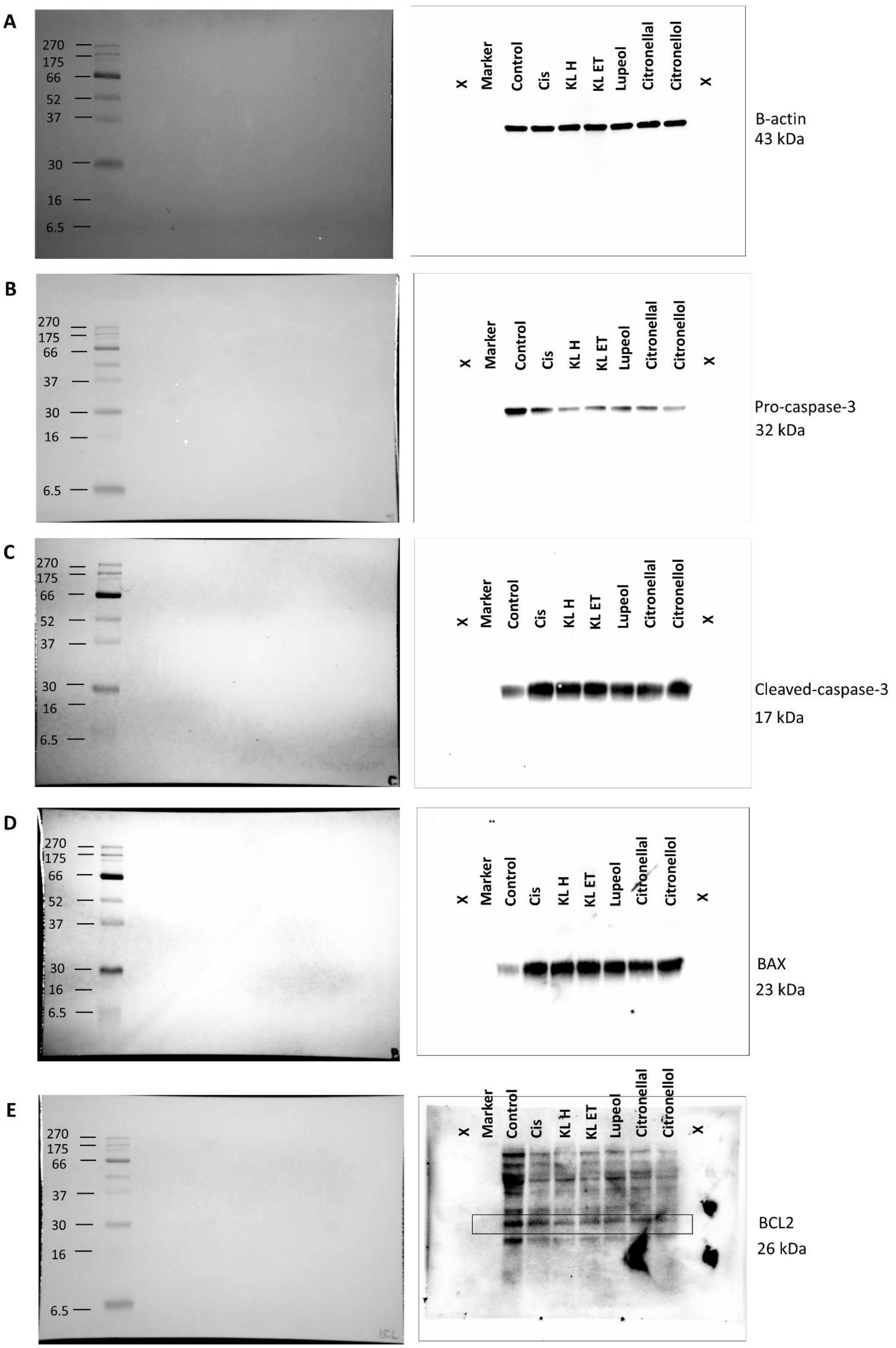
A
270
175
66
52
37
30
16
6.5
X
Marker
Control
Cis
KL H
KL ET
Lupeol
Citronellal
Citronellol
X
B-actin
43 kDa
B
270
175
66
37
30
16
6.5
X
Marker
Control
Cis
KL H
KL ET
Lupeol
Citronellal
Citronellol
X
Pro-caspase-3
32 kDa
C
270
175
66
52
37
30
16
6.5
X
Marker
Control
Cis
KL H
KL ET
Lupeol
Citronellal
Citronellol
X
Cleaved-caspase-3
17 kDa
D
270
175
66
52
37
30
16
6.5
X
Marker
Control
Cis
KL H
KL ET
Lupeol
Citronellal
Citronellol
X
BAX
23 kDa
E
270
175
66
37
30
16
6.5
X
Marker
Control
Cis
KL H
KL ET
Lupeol
Citronellal
Citronellol
X
BCL2
26 kDa

Supplement figure

Full Blots for all Figures. The protein markers were obtained from Bio-Helix Co., LTD. Blultra prestained protein ladder with molecular weights from 6.5 to 270 kDa (Cat.no. PMB01-0500). These images were acquired by ChemiDoc XRS+ Imaging System (Bio-Rad Laboratories Inc., Hercules, CA, USA).
